# Cardiac CEST-MRI for tracking stem cell survival and determining the role of CXCL2

**DOI:** 10.1186/1532-429X-18-S1-P262

**Published:** 2016-01-27

**Authors:** Lina Alon, Dara Kraitchman, Michael Schär, Angel Cortez, Nirbhay N Yadav, Judy Cook, Peter V Johnston, Rebecca Krimins, Michael T McMahon, Peter van Zijl, Jeff W Bulte, Assaf A Gilad

**Affiliations:** 1 grid.21107.350000000121719311Radiology, Johns Hopkins School of Medicine, Baltimore, MD USA; 2 grid.21107.350000000121719311Cellular Imaging Section, Institute for Cell Engineering, Johns Hopkins School of Medicine, Baltimore, MD USA; 3 grid.21107.350000000121719311Center for Image-Guided Animal Therapy, Johns Hopkins School of Medicine, Baltimore, MD USA; 4 grid.240023.7000000040427667XKennedy Krieger Institute, Baltimore, MD USA; 5 grid.21107.350000000121719311Cardiology, Johns Hopkins School of Medicine, Baltimore, MD USA

## Background

Because of the limited endogenous repair mechanisms that exist in the heart, exogenous stem cell therapies offer the potential to provide the building blocks for repair and/or evoke developmental pathways to recruit endogenous cells that can prevent adverse remodeling and promote healing. MR-labeling of stem cells with iron oxides has been used to track stem cells in the heart, but cannot distinguish viable stem cells from cells that have died and released iron oxides. In contradistinction, reporter gene labeling offers the advantage that the reporter is only produced by viable cells. Recently, chemical exchange saturation transfer (CEST) MRI has been performed in the heart (cardioCEST) to endogenously detect fibrosis after myocardial infarction in mice (Vandsburger, M. *et al.Circ Cardiovasc Imaging***8**, (2015)). In this study, we seek to image reporter gene-labeled mesenchymal stem cells (MSCs) in an *in vivo* pig model using cardioCEST MRI.

## Methods

Human MSCs (hMSCs) were transfected with the herpes simplex virus type-1-thymidine kinase (HSV1-tk) reporter gene driven by the CXCL 12 promoter, which is expressed by stem cells and has been shown to facilitate cardiac recovery after myocardial infarction (Penn, M.S. *et al. Gene Ther***19**, 583-87 (2012)). All animal studies were approved by the ACUC. Prior to administration, transfected MSCs were incubated for 3 hours in 5-methyl-5, 6-dihydrothymidine (5-MDHT)(Bar-Shir, A., *et al.Nat. Protocols***8**, 2380-91 (2013)) and then washed. Under general anesthesia with X-ray fluoroscopic guidance, a steerable guide catheter (Myocath, Bioheart) was advanced to the left ventricle (LV) via a femoral approach. Multiple transmyocardial injections of 1. hMSCs, 2. hMSCs incubated with 5-MDHT, and 3. transfected hMSCs incubated with 5-MDCT were performed in the LV septum. Using a 3T MRI (Achieva, Philips), ECG-gated, breath-hold segmented gradient echo (GRE) images were obtained with the following parameters: 32 × 28 cm^2^ FOV; 2 × 2 × 20 mm^3^ voxel; 23 segments/shot; and triggered at end-systole every third heartbeat in the long axis plane. Five sinc-gaussian shaped frequency selective 22000 pulses were applied before data acquisition. Fifty-one different frequencies were saturated from -833 Hz to 833 Hz in steps of 33 Hz to obtain cardioCEST MRIs. CardioCEST MRs were displayed as pseudocolor images for interpretation.

## Results

**Figure 1:**
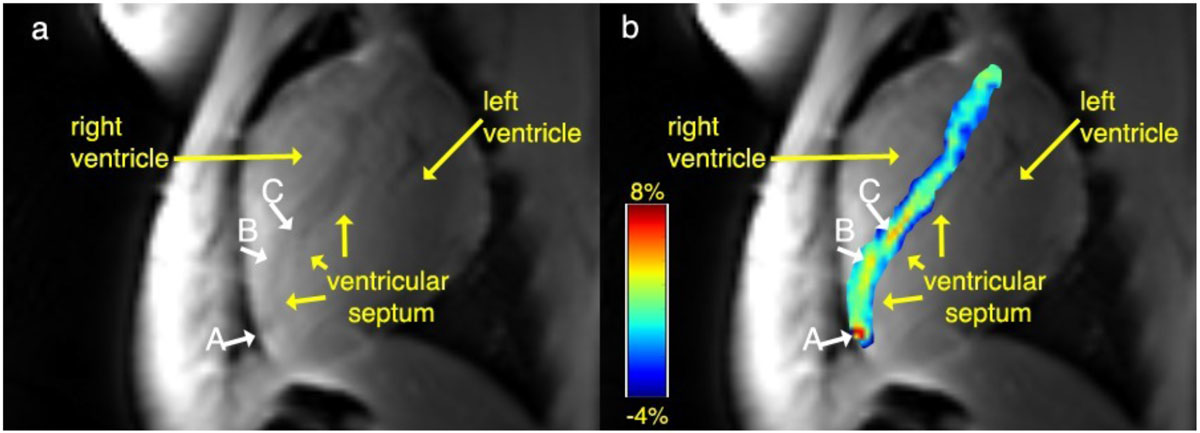
**a| An anatomical MR image of the heart showing the right ventricle, left ventricle, and the interventricular septum where the stem cells were transplanted: MSCs expressing HSV1-TK (A) and wild type MSCs (B) were incubated for four hours with 5-MDHT prior to transplantation and control wild type MSCs (C)**. b| CEST map overlaid on the anatomical image showing labeled MSC^HSV1-TK^ appearing as a hot spot (A).

Average breath-hold time for cardioCEST images was 20 seconds. Representative cardioCEST MRIs are shown in Figure 1. The MSC injection sites were not visible on cine MRIs. cardioCEST MRI was able to distinguish HSV1-tk hMSCs incubated with the reporter probe vs control MSCs.

## Conclusions

These findings indicate the feasibility of imaging gene expression with HSV1-tk and detecting the reporter probe in the heart at clinical field strengths to overcome some of the issues with direct cell labeling techniques. Moreover, the specific absorption rate (SAR) that was used to generate these images was well within clinically allowed limits.

